# Pathogen spectrum of pulmonary infections in kidney transplant recipients and the diagnostic value of mNGS: a sputum and BALF study based on clinical decision-making

**DOI:** 10.3389/fcimb.2026.1742153

**Published:** 2026-05-22

**Authors:** Chao Li, Xin Ye, Yunze Chen, Meili Shen, Zheng Zhou, Hao Jiang, Linkun Hu, Hao Pan, Dan Shen, Yuxin Lin, Liangliang Wang

**Affiliations:** 1 Department of Urology, The First Affiliated Hospital of Soochow University, Suzhou, China; 2 Medical Department, Nanjing Dinfectome Technology Inc., Nanjing, China; 3 Department of Respiratory Medicine, The First Affiliated Hospital of Soochow University, Suzhou, China; 4 Center for Systems Biology, Soochow University, Suzhou, China

**Keywords:** bronchoalveolar lavage fluid, kidney transplantation, metagenomic next-generation sequencing, pathogen spectrum, pulmonary infection, sputum

## Abstract

**Background:**

Pulmonary infection is a common and severe post-transplant complication in kidney transplant recipients (KTRs). Their long-term immunosuppression results in an extremely complex pathogen spectrum. Compared with conventional etiological detection methods, metagenomic next-generation sequencing (mNGS) enables rapid and broad-spectrum pathogen identification. However, compared with bronchoalveolar lavage fluid (BALF), research on the diagnostic value of sputum - used as a non-invasive sample - for pulmonary infections in KTRs remains limited.

**Methods:**

A retrospective study included 77 kidney transplant recipients (KTRs) with pulmonary infections admitted from July 2021 to January 2025. BALF (n=37) or sputum (n=40) was collected for mNGS. Ninety-two non-immunosuppressed patients with pulmonary infections, treated during the same period and with BALF for mNGS, were also included. We compared pathogen profiles between the two groups and evaluated the diagnostic performance for KTRs pulmonary infections between BALF and sputum.

**Results:**

The pathogen spectrum in KTRs was dominated by viruses (43.0%) and opportunistic fungi (20.0%), whereas bacteria (67.97%) predominated in the non-immunosuppressed group. The co-infection rate was significantly higher in KTRs than in the non-immunosuppressed group (67.57% vs. 35.87%, P<0.001). In the KTRs cohort, the sputum group had a much higher prevalence of heart disease than the BALF group (52.5% vs. 2.7%, P<0.001). The positive detection rates of sputum and BALF mNGS showed no statistical difference (97.5% vs. 91.89%, P = 0.268), but sputum mNGShad a higher concordance rate with the clinical composite diagnosis (95.0%) compared to BALF mNGS (81.08%). In both specimen types, mNGS achieved a significantly higher pathogen detection rate than conventional tests (P<0.001 for both), with poor agreement between the two approaches (Kappa < 0.2).

**Conclusion:**

The pathogen spectrum of pulmonary infections in KTRs differs significantly from that in non-immunosuppressed patients. It is characterized by a predominance of viruses and opportunistic fungi. mNGS is superior to conventional methods for making an etiological diagnosis. Non-invasive sputum mNGS is a valuable diagnostic alternative in KTRs, particularly for patients unable or unwilling to undergo invasive procedures.

## 1 Introduction

Kidney transplantation is the most effective therapy for end-stage renal disease, significantly improving long-term survival and quality of life for patients (Hart et al., 2021). However, long-term administration of immunosuppressants significantly increases the risk of opportunistic infections in kidney transplant recipients (KTRs) (Fishman, 2017). Among these, pulmonary infection is one of the most common and life-threatening complications. It often presents with critical illness and poses a significant threat to graft outcomes and patient survival (van Delden et al., 2020; Kamińska et al., 2022).

Rapid and accurate etiological diagnosis is essential for the effective management of pulmonary infections. However, conventional methods—such as smear microscopy and culture—are limited by low sensitivity, long turnaround times, and poor ability to detect co-infections and atypical pathogens (Zheng et al., 2021). These drawbacks often cause diagnostic and therapeutic delays, especially in KTRs, who typically have complex pathogen spectrums and atypical clinical manifestations (Casto et al., 2022). In recent years, metagenomic next-generation sequencing (mNGS), an unbiased high-throughput sequencing technology, has emerged as a powerful tool for diagnosing infectious diseases (Chiu and Miller, 2019; Gu et al., 2019). Previous studies have demonstrated that mNGS can substantially improve pathogen detection rates and provide crucial data to guide personalized clinical therapy (Miller and Chiu, 2021).

Currently, bronchoalveolar lavage fluid (BALF) is considered the “gold standard” specimen for diagnosing pulmonary infections with mNGS (Tao et al., 2022; Zhang et al., 2023; Gudisa et al., 2024). However, its collection is an invasive procedure with procedural risks, limiting its widespread clinical use. Sputum is non-invasive, easy to collect, and enables repeated sampling, but its diagnostic utility has long been debated due to potential contamination from upper respiratory tract commensals (Wang et al., 2015; Zhang et al., 2019). The unbiased, high-throughput nature of mNGS, combined with advanced bioinformatic algorithms, offers the potential to distinguish true pathogens from complex microbial backgrounds, thus highlighting the need to re-evaluate sputum’s diagnostic utility. Nevertheless, data on whether sputum mNGS can match BALF’s diagnostic performance in kidney transplant recipients (KTRs) remains scarce (Chen et al., 2022; Wang et al., 2023). Additionally, research on pathogen spectrum differences between KTRs and non-immunosuppressed individuals is still limited (van Delden et al., 2020; Mahalingasivam et al., 2021). It is crucial to clarify these differences to optimise diagnostic algorithms and guide empirical therapeutic strategies.

Therefore, this study aims to comprehensively characterize the differences in the pathogen spectrum of pulmonary infections between KTRs and non-immunosuppressed patients using mNGS. Concurrently, based on real-world clinical data, we seek to evaluate the clinical utility of sputum mNGS in KTRs, with a particular focus on its diagnostic potential for patients unable to tolerate invasive sampling. The findings are intended to provide evidence for optimizing rapid, precise diagnostic pathways for post-kidney transplantation pulmonary infections.

## 2 Materials and methods

### 2.1 Study design and participants

This retrospective study enrolled kidney transplant recipients (KTRs) and non-immunosuppressed controls who were diagnosed with pulmonary infection at the First Affiliated Hospital of Soochow University between July 2021 and January 2025. Pulmonary infection was diagnosed based on combined radiological and clinical criteria (Shi and Zhu, 2021). Radiological criteria included new or progressive infiltrates on chest X-ray or CT. Clinical criteria required meeting at least two of the following: (1) fever (body temperature ≥ 38 °C); (2) cough and/or sputum production, or worsening of pre-existing respiratory symptoms; (3) leukocytosis (white blood cell count ≥ 10.0 × 10^9^/L); (4) clinical signs of pulmonary consolidation and/or audible moist rales on auscultation.

A total of 77 KTRs with post-transplant pulmonary infection were included from the Department of Urology. Concurrently, 92 non-immunosuppressed patients diagnosed with community-acquired or hospital-acquired pneumonia and without any known immunodeficiency were recruited from the Department of Respiratory Medicine as controls. We collected both mNGS and conventional microbiological testing data from sputum and BALF specimens in the KTR cohort, and additionally gathered mNGS data from BALF specimens in the non-immunosuppressed cohort. Based on clinically collected specimens, KTRs were further stratified into two subgroups: the BALF group (n=37) and the sputum group (n=40). The inclusion criteria for the KTRs group were: (1) age ≥18 years; (2) recipient of an allogeneic kidney transplant; (3) clinically and radiologically confirmed pulmonary infection; and (4) completion of mNGS testing on either BALF or sputum specimen, in addition to conventional etiological tests. The inclusion criteria for the non-immunosuppressed group were: (1) age ≥18 years; (2) clinically and radiologically confirmed pulmonary infection; (3) no history of immunodeficiency or immunosuppressive agents use (e.g., organ transplantation, HIV infection, active malignancy, autoimmune disease, long-term or recent use of immunosuppressants or corticosteroids); and (4) completion of mNGS testing on BALF. The exclusion criteria were: (1) missing or incomplete mNGS results; (2) concurrent infections at other sites; (3) unqualified specimens or those failing mNGS quality control; and (4) incomplete clinical data.

The study was performed in accordance with the ethical principles of the Declaration of Helsinki and was approved by the Ethics Committee of the First Affiliated Hospital of Soochow University (Approval No. 20251056). The requirement for informed consent was waived by the ethics committee. All kidneys were procured from deceased citizens or living-related donors. All patient data were extracted from electronic medical records and anonymized prior to analysis.

### 2.2 Clinical data collection

The following data were collected and recorded for each patient: demographic characteristics (age, sex), clinical manifestations (temperature, length of hospital stay, symptoms), baseline comorbidities (e.g., hypertension, diabetes, heart disease), previous pulmonary infection (pulmonary infection occurring within 1 year before the current infection), infection type (single or mixed), and antimicrobial treatment regimens and outcomes. For all enrolled KTRs, tacrolimus trough concentrations and serum creatinine levels were collected at two time points: admission (before the initiation of antimicrobial therapy) and discharge (after the completion of standardized antimicrobial treatment). For the classification of antimicrobial regimen adjustments based on mNGS results, standardized and objective definitions were pre-specified as follows: (1) De-escalation: Reduction in the antimicrobial spectrum, number of antimicrobial agents, or step-down from broad-spectrum to narrow-spectrum agents, guided by mNGS etiological results. (2)Initiation: *De novo* initiation of targeted antimicrobial treatment that was not administered prior to mNGS testing. (3) Escalation: Addition of new antimicrobial classes, expansion of antimicrobial coverage spectrum, or increase in therapeutic intensity based on mNGS results. (4) Maintained: Continuation of the original antimicrobial regimen without any modification, as mNGS results supported the appropriateness of the ongoing treatment. (5) Switch: Replacement of one antimicrobial agent with another agent of similar antimicrobial spectrum and therapeutic intensity, without escalation or de-escalation of coverage. All regimen classifications were independently assessed by two senior physicians blinded to mNGS results, with discrepancies resolved by a third senior infectious disease specialist to minimize subjectivity. All data were independently entered and cross-checked by two researchers.

### 2.3 Specimen colection and conventional etiological tests

BALF specimens were collected by experienced pulmonologists via bronchoscopy under sterile conditions. Sputum specimens required deep-cough collection, with quality confirmed by microscopy (≤10 squamous epithelial cells per low-power field). All specimens were transported to the laboratory immediately. One aliquot was used for conventional etiological tests, including smears, bacterial/fungal cultures, galactomannan (GM) test, (1,3)-β-D-glucan (G) test, and pathogen-specific PCR assays. The other aliquot (approximately 1.5–2 mL) was used for mNGS.

### 2.4 Metagenomic next-generation sequencing

Sputum and viscous BALF samples were liquefied by 0.1% dithiothreitol (DTT) for 20 minutes at 56°C before extraction. Then DNA was extracted using the TIANamp Magnetic DNA Kit (TIANGEN, China), according to the manufacturer’s protocol. The quantity and quality of DNA were evaluated using Qubit 2.0 Fluorometers and Nanodrop 8000 spectrophotometers (Thermo Fisher Scientific, USA), respectively. DNA library construction was performed according to the instructions of Hieff NGS C130P2 OnePot II DNA Library Prep Kit for MGI (Yeasen Biotech, Shanghai, China). The Agilent 2100 Bioanalyzer system (Agilent, USA) and the Qubit dsDNA HS Assay Kit (Thermo Fisher Scientific, USA) was used to control the DNA library fragment sizes and concentrations. Finally, we completed sequencing in the single-end 50 bp sequencing mode using DIFSEQ-200 (Dinfectome, China). NTCs were also included in the library preparation and sequencing process.

### 2.5 Bioinformatic analysis

Raw sequencing data were splited by bcl2fastq2 (version 2.20), and high-quality sequencing data were generated using Trimmomatic (version 0.36) by removing low quality reads, adapter contamination, duplicated and shot (length<36 bp) reads. Human host sequence were subtracted by mapping to human reference genome (hs37d5) using bowtie2 (version 2.2.6). Reads that could not be mapped to the human genome were retained and aligned with microorganism genome database for microbial identification by Kraken (version 2.0.7), and for species abundance estimating by Bracken (version 2.5.0). The microorganism genome database contained genomes or scaffolds of bacteria, fungi, viruses and parasites (download from GenBank release 238, http://ftp.ncbi.nlm.nih.gov/genomes/genbank/).

We used the following criteria when interpreting the results of mNGS: For *Mycobacterium*, *Nocardia*, and *Legionella pneumophila*, a positive result was defined when a species-specific read count of 1 or more was detected by mNGS. For bacteria (excluding *Mycobacterium*, *Nocardia*, and *Legionella pneumophila*), fungi, viruses, and parasites, a positive result was defined when a species had at least three non-overlapping reads detected by mNGS. Pathogens detected in the negative NTCs were excluded from the analysis, except when the read counts in the samples were at least 10-fold higher than those observed in the NTCs.

### 2.6 Key definitions and clinical assessment

Clinical composite diagnosis (reference standard): The final etiological diagnosis was independently determined by two senior physicians blinded to mNGS results. This composite diagnosis was based on: (1) alignment of clinical syndrome with a specific pathogen; (2) radiological findings; (3) conventional etiological test results; and (4) clinical response to targeted antimicrobial therapy (Yang et al., 2022). A positive clinical response was defined as definite improvement in clinical signs (e.g., fever resolution, reduced cough/sputum), symptoms (e.g., fewer rales), and inflammatory markers (e.g., >50% decrease in C-reactive protein or procalcitonin level from baseline) within 72 to 96 hours after initiating targeted therapy based on mNGS-identified pathogens. Discrepancies between the two physicians were resolved by discussion. if consensus was not reached, a senior infectious disease specialist provided final adjudication. For co-infection, the primary pathogen was defined as the microorganism best supported by multiple lines of evidence.

Diagnostic accuracy metrics: By comparing with the reference standard, mNGS results were categorized as true positive (TP), false positive (FP), true negative (TN), or false negative (FN).

Assessment of mNGS clinical impact: The clinical impact of mNGS was categorized as “positive” or “negative” by comparing its results with the reference standard. “Positive” impact was assigned if mNGS results were fully concordant with the clinical composite diagnosis, or provided critical etiological evidence that guided subsequent effective targeted antimicrobial therapy. Example: mNGS detected Human cytomegalovirus (CMV) and Pneumocystis jirovecii in a KTR with pulmonary infection. Targeted anti-CMV and anti-Pneumocystis therapy was initiated immediately based on mNGS results, and the patient achieved significant improvement in fever, respiratory symptoms, and inflammatory markers (≥50% reduction in C-reactive protein) within 72 hours, which was fully consistent with the final clinical composite diagnosis.

“Negative” impact was assigned if mNGS results were discordant with the clinical composite diagnosis, only detected upper respiratory commensals without clinical or radiological support, or failed to provide clinically actionable etiological information. Example: mNGS only detected Candida albicans (a common upper respiratory tract commensal) in a KTR, with no corresponding clinical manifestations, radiological abnormalities, or positive conventional test results to support pathogenicity. This result did not guide any clinical treatment adjustment.

### 2.7 Statistical analysis

Statistical analyses were performed using SPSS version 27.0. Continuous data with a normal distribution were presented as mean ± standard deviation and compared using the t-test. Non-normally distributed continuous data were presented as median (interquartile range, IQR) and compared using the Mann-Whitney U test. Categorical data were presented as frequency (percentage) and compared using the Chi-square test or Fisher’s exact test. Using the clinical composite diagnosis as the reference standard, The sensitivity, specificity, positive predictive value (PPV), negative predictive value (NPV), and accuracy of mNGS were calculated for different specimen types. Cohen’s Kappa coefficient was used to assess the concordance between mNGS and conventional tests. A two-sided P-value < 0.05 was considered statistically significant.

## 3 Results

### 3.1 Comparison of pathogen spectrum between kidney transplant recipients and non-immunosuppressed patients

#### 3.1.1 Study population and baseline characteristics

37 KTRs and 92 non-immunosuppressed patients with pulmonary infections who underwent BALF mNGS were enrolled. Detailed baseline characteristics are presented in **Table 1**. No significant differences were observed in age or body temperature between the two groups (P > 0.05). However, KTRs had a significantly longer length of hospital stay than the non-immunosuppressed group (median: 12 vs. 9 days, P = 0.007) and a markedly higher prevalence of hypertension (91.89% vs. 29.35%, P < 0.001) and co-infections (67.57% vs. 35.87%, P < 0.001). Regarding clinical symptoms, KTRs were more likely to present with fever (89.19% vs. 63.04%, P = 0.003) and fatigue (27.03% vs. 10.87%, P = 0.022). Conversely, sputum production was more prevalent among non-immunosuppressed patients (77.17% vs. 54.05%, P = 0.009).

**Table 1:** Comparison of baseline characteristics between KTRs and non-immunosuppressed patients.

Characteristic	Category	KTRs (n=37)	Non-immunosuppressed (n=92)	P
Age (years), median (IQR)		50 (42, 56)	54.5 (38.25, 67)	0.187
Temperature (°C), median (IQR)		38 (37.55, 38.5)	37.8 (37, 38.9)	0.285
Length of hospital stay (days), median (IQR)		12 (9.5, 15.5)	9 (7, 13.75)	0.007
Sex, n (%)	Male	19 (51.35%)	37 (40.22%)	0.249
	Female	18 (48.65%)	55 (59.78%)	
Comorbidities, n (%)	Pulmonary disease	0 (0.0%)	8 (8.7%)	0.064
	Hypertension	34 (91.89%)	27 (29.35%)	<0.001
	Hyperuricemia	1 (2.7%)	1 (1.09%)	0.493
	Diabetes	7 (18.92%)	12 (13.04%)	0.394
	Heart disease	1 (2.7%)	6 (6.52%)	0.672
	Uremia	0 (0.0%)	1 (1.09%)	0.524
	None	2 (5.41%)	54 (58.7%)	<0.001
Clinical Symptoms, n (%)	Fever	33 (89.19%)	58 (63.04%)	0.003
	Fatigue	10 (27.03%)	10 (10.87%)	0.022
	Cough	32 (86.49%)	84 (91.3%)	0.411
	Sputum production	20 (54.05%)	71 (77.17%)	0.009
	Dyspnea	10 (27.03%)	32 (34.78%)	0.395
	Headache	0 (0.0%)	1 (1.09%)	0.524
	Chest tightness	21 (56.76%)	41 (44.57%)	0.210
	Dizziness	1 (2.7%)	0 (0.0%)	0.113
Infection Type, n (%)	Co-infection	25 (67.57%)	33 (35.87%)	<0.001
	Mono-infection	9 (24.32%)	59 (64.13%)	
	Not detected	3 (8.11%)	0 (0.0%)	

#### 3.1.2 Pathogen profile characteristics in KTRs and non-immunosuppressed patients based on mNGS

**Figure 1:**
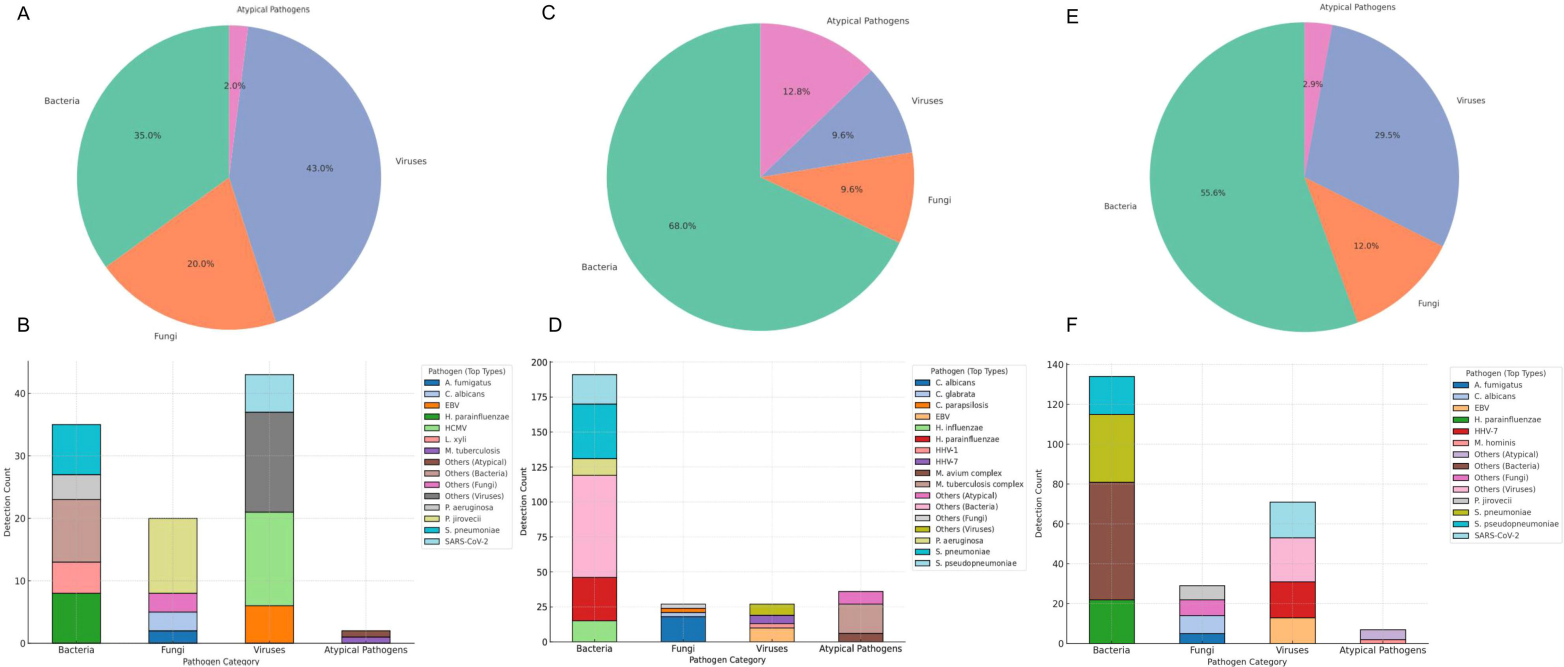
Distribution of pathogen categories detected by mNGS in KTRs and non-immunosuppressed patients. The pie chart displays the proportion of different pathogen categories among all detected pathogens by BALF mNGS in KTRs **(A)**, and non-immunosuppressed patients **(C)**, and by sputum mNGS in KTRs **(E)**. Stacked bar chart of detected pathogens by category by BALF mNGS in KTRs **(B)**, non-immunosuppressed patients **(D)**, and by sputum mNGS in KTRs **(F)**.

BALF mNGS revealed fundamental differences in the pathogen spectrum between KTRs and non-immunosuppressed patients. In the KTRs group (n=37, 100 total detections), viruses (43.0%) were the predominant pathogen category, with human cytomegalovirus (CMV, 15.0%), *Epstein-Barr virus* (EBV, 6.0%), and Severe Acute Respiratory Syndrome Coronavirus 2 (SARS-CoV-2, 6.0%) being the most frequent. Bacteria accounted for 35.0% of the detections, with *Streptococcus pneumoniae* (8.0%) and *Haemophilus parainfluenzae* (8.0%) being the most prevalent species. Fungi constituted 20.0% of detections, with *Pneumocystis jirovecii* (12.0%) being the dominant species. Atypical pathogens were detected at a lower rate of 2.0% (**Table 2**; **Figures 1A, B**). In sharp contrast, the pathogen spectrum of non-immunosuppressed patients (n=92, 281 total detections) was dominated by bacteria (67.97%). The main bacterial species were still *Streptococcus pneumoniae* (13.88%) and *Haemophilus parainfluenzae* (11.03%). Atypical pathogens were detected at a significantly higher proportion (12.81%), led by *Mycobacterium tuberculosis* complex (7.47%). Viruses (9.61%) and fungi (9.61%) were relatively uncommon, with EBV and *Candida albicans* being the most frequently identified, respectively (**Table 3**; **Figures 1C, D**). The detection rate of *Mycobacterium* species in the non−immunosuppressed group was significantly higher than in the KTR group (12.81% vs 2.00%).

**Table 2:** Pathogens detected by mNGS in BALF specimens from kidney transplant recipients.

Category	Pathogen	Total detections	Proportion (%)
Bacteria		35	35.00
	*Streptococcus pneumoniae*	8	8.00
	*Haemophilus parainfluenzae*	8	8.00
	*Corynebacterium striatum*	5	5.00
	*Pseudomonas aeruginosa*	4	4.00
	*Staphylococcus aureus*	2	2.00
	*Proteus mirabilis*	2	2.00
	*Streptococcus pseudopneumoniae*	2	2.00
	*Stenotrophomonas maltophilia*	2	2.00
	*Tropheryma whipplei*	1	1.00
	*Acinetobacter baumannii complex*	1	1.00
Fungi		20	20.00
	*Pneumocystis jirovecii*	12	12.00
	*Candida albicans*	3	3.00
	*Aspergillus fumigatus*	2	2.00
	*Cladosporium sphaerospermum*	1	1.00
	*Aspergillus terreus*	1	1.00
	*Scedosporium boydii*	1	1.00
Viruses		43	43.00
	Human cytomegalovirus (CMV)	15	15.00
	*Epstein-Barr virus* (EBV)	6	6.00
	*Human alphaherpesvirus 1* (HSV-1)	2	2.00
	*Human betaherpesvirus 7* (HHV-7)	2	2.00
	*Human mastadenovirus B*	1	1.00
	*Hepatitis B virus* (HBV)	1	1.00
	SARS-CoV-2	6	6.00
	*Human parainfluenza virus 3* (HPIV-3)	2	2.00
	*Human coronavirus 229E* (HCoV-229E)	1	1.00
	*Betacoronavirus 1* (HCoV-OC43)	1	1.00
	*Human orthopneumovirus* (RSV)	2	2.00
	*Influenza B virus* (FluB)	1	1.00
	*Influenza A virus* (FluA)	1	1.00
	*Rhinovirus A*	1	1.00
	*Human metapneumovirus*	1	1.00
Atypical Pathogens		2	2.00
	*Mycoplasma pneumoniae*	1	1.00
	*Mycobacterium tuberculosis* complex	1	1.00

**Table 3:** Pathogens detected by mNGS in BALF specimens from non-immunosuppressed patients.

Category	Pathogen	Total detections	Proportion (%)
Bacteria		191	67.97
	*Streptococcus pneumoniae*	39	13.88
	*Haemophilus parainfluenzae*	31	11.03
	*Streptococcus pseudopneumoniae*	21	7.47
	*Haemophilus influenzae*	15	5.34
	*Pseudomonas aeruginosa*	12	4.27
	*Corynebacterium striatum*	12	4.27
	*Staphylococcus aureus*	9	3.20
	*Klebsiella pneumoniae*	7	2.49
	*Enterococcus faecium*	5	1.78
	*Stenotrophomonas maltophilia*	5	1.78
	*Finegoldia magna*	5	1.78
	*Enterococcus faecalis*	4	1.42
	*Acinetobacter baumannii complex*	3	1.07
	*Achromobacter xylosoxidans*	2	0.71
	*Haemophilus parahaemolyticus*	2	0.71
	*Tropheryma whipplei*	2	0.71
	*Escherichia coli*	2	0.71
	*Morganella morganii*	2	0.71
	*Moraxella catarrhalis*	2	0.71
	*Enterococcus gallinarum*	1	0.36
	*Legionella pneumophila*	1	0.36
	*Leuconostoc citreum*	1	0.36
	*Streptococcus pyogenes*	1	0.36
	*Actinomyces odontolyticus*	1	0.36
	*Pasteurella multocida*	1	0.36
	*Nocardia transvalensis*	1	0.36
	*Nocardia anonyma*	1	0.36
	*Nocardia mexicana*	1	0.36
	*Stenotrophomonas maltophilia group*	1	0.36
	*Enterococcus hirae*	1	0.36
Fungi		27	9.61
	*Candida albicans*	18	6.41
	*Candida tropicalis*	1	0.36
	*Candida parapsilosis*	3	1.07
	*Candida glabrata*	3	1.07
	*Aspergillus fumigatus*	1	0.36
	*Aspergillus oryzae*	1	0.36
Viruses		27	9.61
	*Epstein-Barr virus* (EBV)	10	3.60
	*Human betaherpesvirus 7* (HHV-7)	6	2.14
	*Human alphaherpesvirus 1* (HSV-1)	3	1.07
	*Human betaherpesvirus 6B* (HHV-6B)	1	0.36
	Human cytomegalovirus (CMV)	2	0.71
	*Human coronavirus HKU1*	1	0.36
	*Human mastaden*ovirus C	1	0.36
	*β-papillomavirus 1*	2	0.71
	*β-papillomavirus 3*	1	0.36
Atypical Pathogens		36	12.81
	*Mycobacterium tuberculosis* complex	21	7.47
	*Mycobacterium avium* complex	6	2.14
	*Mycobacterium kansasii*	2	0.71
	*Mycobacterium abscessus*	1	0.36
	*Mycoplasma pneumoniae*	2	0.71
	*Mycoplasma salivarium*	1	0.36
	*Chlamydia psittaci*	1	0.36
	*Chlamydia pneumoniae*	1	0.36
	*Rickettsia felis*	1	0.36

These results indicate that the immunosuppressive state profoundly alters the microbial ecology of pulmonary infections, shifting from a bacteria-dominant pattern in immunocompetent hosts to a profile characterized by viruses and opportunistic fungi in KTRs.

### 3.2 Comparison of diagnostic performance between sputum and BALF mNGS

#### 3.2.1 Study population and baseline characteristics

We further analyzed the diagnostic utility of mNGS in the BALF group (n=37) and sputum group (n=40) within the cohort of 77 KTRs. Detailed clinical characteristics of the sputum and BALF groups are presented in **Table 4**. The two groups showed no significant differences in age, temperature, length of hospital stay, or time from transplantation to infection (P > 0.05). However, the time to sampling was significantly longer for the BALF group compared to the sputum group (median 2 vs. 1 day, P<0.001), while no significant difference was observed in the turnaround time to results (P = 0.162). A significant disparity was observed in the prevalence of heart disease, which was markedly lower in the BALF group than in the sputum group (2.7% vs. 52.5%, P<0.001). The distribution of other comorbidities, including hypertension, hyperuricemia and diabetes, was similar between the groups (P>0.05). For clinical symptoms, sputum production was significantly more common in the sputum group (82.5% vs. 54.05%, P = 0.007), while the distribution of other symptoms was comparable between groups. The co-infection rate was 67.57% in the BALF group and 95.0% in the sputum group, with a statistically significant difference (P = 0.007). The use of antibiotic and antiviral agents was comparable between the two groups, however the BALF group had a much higher rate of antifungal agent use (75.68% vs. 32.5%, P<0.001). No significant differences were found in therapy and regimen adjustments based on mNGS results, or patient outcomes, between the two groups.

**Table 4:** Comparison of clinical characteristics between sputum and BALF groups in kidney transplant recipients.

Characteristic	Category	BALF group (n=37)	Sputum group (n=40)	P
Age (years), mean ± SD		49.65 ± 9.17	49.1 ± 10.63	0.81
Temperature (°C), mean ± SD		38.06 ± 0.79	38.13 ± 0.77	0.681
Length of stay (days), median (IQR)		12 (9.5 15.5)	15.5 (8, 22.75)	0.312
Time to infection post-transplant (days), median (IQR)		760 (346, 2190)	730 (217.5, 2098.75)	0.61
Time to sampling (days), median (IQR)		2 (2, 3)	1 (1, 1)	<0.001
Time to result (days), median (IQR)		1 (1, 2)	1 (1, 1)	0.162
Sex, n (%)	Male	19 (51.35%)	23 (57.5%)	0.588
	Female	18 (48.65%)	17 (42.5%)	
Comorbidities, n (%)	Pulmonary disease	0 (0.0%)	2 (5.0%)	0.168
	Hypertension	34 (91.89%)	34 (85.0%)	0.347
	Hyperuricemia	1 (2.7%)	4 (0.0%)	0.194
	Diabetes	7 (18.92%)	7 (17.5%)	0.872
	Heart disease	1 (2.7%)	21 (52.5%)	<0.001
	None	2 (5.41%)	4 (10.0%)	0.452
Previous pulmonary infection within 1 year, n (%)		7 (18.9)	9 (22.5)	0.769
Clinical Symptoms, n (%)	Fever	33 (89.19%)	33 (82.5%)	0.402
	Fatigue	10 (27.03%)	7 (17.5%)	0.314
	Cough	32 (86.49%)	34 (85.0%)	0.852
	Sputum production	20 (54.05%)	33 (82.5%)	0.007
	Dyspnea	10 (27.03%)	11 (27.5%)	0.963
	Chest tightness	21 (56.76%)	16 (40.0%)	0.141
	Dizziness	1 (2.7%)	0 (0.0%)	0.295
Tacrolimus trough concentration at admission (ng/ml)		5.16 (3.99, 6.72)	6.47 (5.21, 6.92)	0.051
Tacrolimus trough concentration at discharge (ng/ml)		4.51 (2.58, 6.05)	4.82 (3.49, 6.04)	0.752
Serum creatinine before admission (μmol/L)		136.30 (114.70, 193.50)	136.00 (85.25, 210.58)	0.400
Serum creatinine after discharge (μmol/L)		138.50 (114.40, 190.80)	138.50 (114.40, 190.80)	0.221
Infection Type, n (%)	Co-infection	25 (67.57%)	38 (95.0%)	0.007
	Mono-infection	9 (24.32%)	1 (2.5%)	
	Not detected	3 (8.11%)	1 (2.5%)	
Antibiotic use, n (%)	Yes	37 (100.0%)	39 (97.5%)	0.333
	No	0(0.0%)	1 (2.5%)	
Antifungal use, n (%)	Yes	28(75.68%)	13 (32.5%)	<0.001
	No	9(24.32%)	27 (67.5%)	
Antiviral use, n (%)	Yes	12(32.43%)	19 (47.5%)	0.178
	No	25(67.57%)	21 (52.5%)	
Therapy adjustment based on mNGS, n (%)	Yes	26(70.27%)	27 (67.5%)	0.793
	No	11(29.73%)	13 (32.5%)	
Medication regimen, n (%)	De-escalation	4 (10.81%)	4 (10.0%)	0.993
	Initiation	7(18.92%)	7 (17.5%)	
	Escalation	13(35.14%)	16 (40.0%)	
	Maintained	12(32.43%)	13 (32.5%)	
	Switched	1(2.7%)	0 (0.0%)	
Outcome, n (%)	Improved	36 (97.30)	39 (97.50)	0.367
	Stable	1 (2.70)	0 (0.00)	
	Death	0 (0.00)	1 (2.50)	

#### 3.2.2 Pathogen spectrum in the sputum group

Sputum mNGS yielded a total of 241 pathogen detections. The detailed distribution is shown in **Table 5**; **Figures 1E, F**. The main pathogen categories were bacteria (55.6%) and viruses (29.5%), with the top detected species being *Streptococcus pneumoniae* (14.11%), *Haemophilus parainfluenzae* (9.13%), SARS-CoV-2 (7.47%), and *Human herpesvirus 7* (HHV-7, 7.47%). Fungi (12.0%) and atypical pathogens (2.9%) made up smaller shares.

**Table 5:** Pathogens detected by mNGS in the sputum group of kidney transplant recipients.

Category	Pathogen	Total detections	Proportion (%)
Bacteria		134	55.60
	*Enterococcus faecalis*	6	2.49
	*Haemophilus parainfluenzae*	22	9.13
	*Streptococcus pneumoniae*	34	14.11
	*Streptococcus pseudopneumoniae*	19	7.88
	*Haemophilus parahaemolyticus*	3	1.24
	*Haemophilus influenzae*	5	2.07
	*Enterococcus avium*	2	0.83
	*Eikenella corrodens*	2	0.83
	*Staphylococcus aureus*	4	1.66
	*Escherichia coli*	1	0.41
	*Acinetobacter baumannii* complex	2	0.83
	*Klebsiella pneumoniae*	4	1.66
	*Finegoldia magna*	5	2.07
	*Streptococcus agalactiae*	1	0.41
	*Klebsiella aerogenes*	1	0.41
	*Staphylococcus argenteus*	1	0.41
	*Tropheryma whipplei*	1	0.41
	*Clostridioides difficile*	2	0.83
	*Burkholderia cepacia* complex	1	0.41
	*Pseudomonas aeruginosa*	4	1.66
	*Klebsiella oxytoca*	1	0.41
	*Corynebacterium striatum*	2	0.83
	*Achromobacter xylosoxidans*	1	0.41
	*Morganella morganii*	1	0.41
	*Leuconostoc citreum*	1	0.41
	*Enterococcus faecium*	2	0.83
	*Nocardia pinae*	1	0.41
	*Acinetobacter baumannii*	2	0.83
	*Enterococcus hirae*	1	0.41
	*Stenotrophomonas maltophilia*	1	0.41
	*Leuconostoc suis*	1	0.41
Fungi		29	12.03
	*Candida albicans*	9	3.73
	*Cryptococcus neoformans/gattii*	1	0.41
	*Pneumocystis jirovecii*	7	2.90
	*Aspergillus fumigatus*	5	2.07
	*Candida tropicalis*	1	0.41
	*Penicillium digitatum*	5	2.07
	*Candida parapsilosis*	1	0.41
Viruses		71	29.46
	*Human parainfluenza virus 3*	1	0.41
	*Human metapneumovirus*	2	0.83
	SARS-CoV-2	18	7.47
	*Human alphaherpesvirus 1*	7	2.90
	*Epstein-Barr virus*	13	5.39
	*Influenza B virus*	1	0.41
	*Rhinovirus A*	2	0.83
	*Influenza A virus*	1	0.41
	*Human betaherpesvirus 7*	18	7.47
	*Human betaherpesvirus 6A*	1	0.41
	*Rhinovirus C*	1	0.41
	*Primate erythroparvovirus 1*	1	0.41
	*Human cytomegalovirus*	3	1.24
	*β-papillomavirus 1*	1	0.41
	*Human betaherpesvirus 6B*	1	0.41
Atypical Pathogens		7	2.90
	*Mycoplasma pneumoniae*	1	0.41
	*Mycoplasma hominis*	2	0.83
	*Ureaplasma parvum*	1	0.41
	*Mycobacterium abscessus*	1	0.41
	*Mycobacterium fortuitum*	1	0.41
	*Mycoplasma fermentans*	1	0.41

Compared with mNGS results in BALF, sputum mNGS exhibited certain differences in pathogen spectrum detection. For bacterial detection, sputum mNGS showed a higher detection rate (55.6% vs 35.0% in BALF), with common lower respiratory tract pathogens such as Streptococcus pneumoniae and Haemophilus parainfluenzae as the predominant detected species. In contrast, for opportunistic pathogens that colonize and replicate in alveolar epithelial cells, including human cytomegalovirus (CMV, 1.24% vs 15.0% in BALF) and Pneumocystis jirovecii (2.9% vs 12.0% in BALF), the detection rate of sputum mNGS was significantly lower than that of BALF mNGS. No significant difference was observed in the detection rate of Epstein−Barr virus (EBV, 5.39% vs 6.0% in BALF) between the two specimen types. (**Tables 2**, **5**).

#### 3.2.3 Sputum mNGS demonstrates good diagnostic accuracy

**Figure 2:**
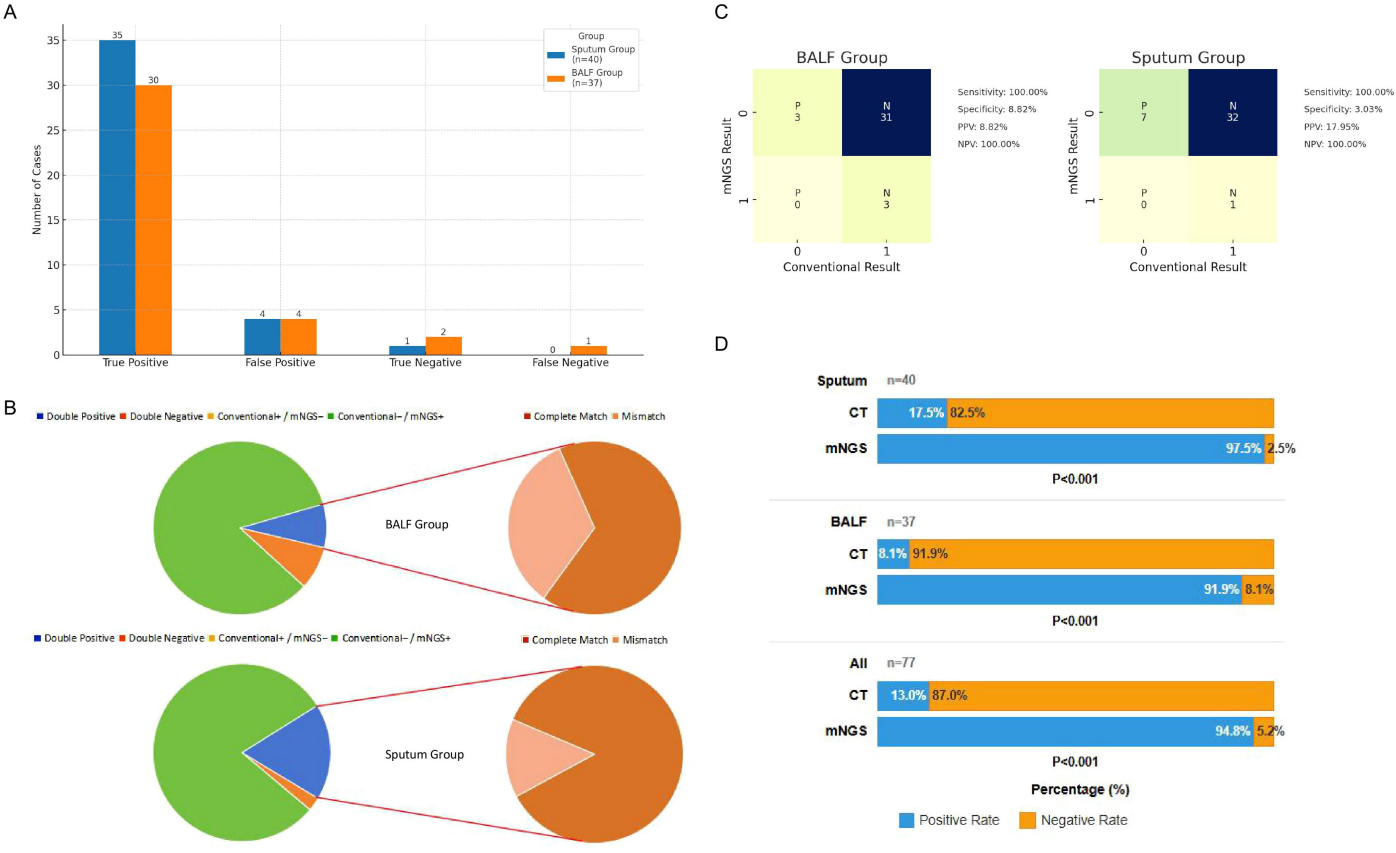
The Comparison of Performance among the Sputum, BALF mNGS and Conventional Tests. **(A)** Bar chart of diagnostic consistency of sputum and BALF mNGS groups. **(B)** Concordance analysis between mNGS and conventional tests in the BALF and sputum groups. **(C)** Heatmap of the consistency between mNGS and conventional tests in the BALF and sputum groups. The x-axis represents the conventional test results (0 = negative, 1 = positive), and the y-axis represents the mNGS result (P = positive, N = negative). **(D)** Comparison of positive rates between mNGS and conventional tests in the BALF and sputum groups. PPV, positive predictive value; NPV, negative predictive value; BALF, bronchoalveolar lavage fluid; CT, conventional tests.

The comparison of performance between the sputum and BALF mNGS groups is shown in **Table 6** and **Figure 2**. Both sputum and BALF mNGS achieved positive detection rates over 90%, with no significant inter-group difference (97.5% vs. 91.89%, P = 0.268). Using the composite clinical diagnosis as the reference standard, there were no significant differences in the true positive (TP) rate, false positive (FP) rate, true negative (TN) rate, or false negative (FN) rate between the two groups (**Figure 2A**). However, the positive clinical impact rate of sputum mNGS was higher than that of the BALF group (95.0% vs. 81.08%, P = 0.058). These findings indicate that sputum mNGS has good diagnostic performance for pulmonary infections in kidney transplant recipients, and its performance is comparable to that of BALF mNGS, or even superior.

**Table 6:** Comparison of positive concordance, clinical impact and diagnostic accuracy between sputum and BALF mNGS groups.

Characteristic	Category	Sputum group (n=40)	BALF group (n=37)	P-value
Positive Concordance Rate	Concordant	39 (97.5%)	34 (91.89%)	0.268 (Fisher’s exact P = 0.346)
	Discordant	1 (2.5%)	3 (8.11%)	
Clinical Impact of mNGS	Positive	38 (95.0%)	30 (81.08%)	0.058
	Negative	2 (5.0%)	7 (18.92%)	
Pathogen Identification	True Positive	35 (87.5%)	30 (81.08%)	0.659
	False Positive	4 (10.0%)	4 (10.81%)	
	True Negative	1 (2.5%)	2 (5.41%)	
	False Negative	0 (0.0%)	1 (2.7%)	

The concordance metrics in this table are calculated using the clinical composite diagnosis as the reference standard. Positive concordance rate = (Number of cases with consistent positive results between mNGS and clinical composite diagnosis)/Total number of cases × 100%).

#### 3.2.4 Advantages of mNGS over conventional tests

For both BALF and sputum specimens, the concordance between mNGS and conventional tests (CT) was extremely low (Kappa < 0.2 for both, **Table 7**). In the vast majority of cases where mNGS was positive, CT were negative (BALF group: 31/34 [91.2%], sputum group: 32/39 [82.1%], **Figure 2B**). The sensitivity of mNGS reaches up to 100% for both BALF and sputum specimens (**Figure 2C**), with a significantly higher positive rate than conventional tests (**Figure 2D**). This underscores the remarkable sensitivity of mNGS in detecting low-biomass pathogens, atypical pathogens, and co-infections.

**Table 7:** Detection rates and concordance between mNGS and conventional methods.

Characteristic	Category	Conventional positive (n)	Conventional negative (n)	P-value	Kappa value
BALF Group (n=37)	mNGS Positive (n)	3	31	1	0.015
	mNGS Negative (n)	0	3		
Sputum Group (n=40)	mNGS Positive (n)	7	32	1	0.011
	mNGS Negative (n)	0	1		

Concordance in this table is assessed between mNGS and conventional etiological tests, calculated using Cohen’s Kappa coefficient. Kappa values < 0.2 indicate extremely low agreement between mNGS and conventional testing methods, with results showing significant discrepancy.

## 4 Discussion

In the complex field of post-transplant infectious diseases, precise etiological diagnosis remains a pivotal challenge to improving patient outcomes. This study systematically compared the pathogen profiles of pulmonary infections in kidney transplant recipients (KTRs) versus non-immunosuppressed patients using mNGS and evaluated the diagnostic performance of sputum versus BALF specimens in the KTR population. Our findings reveal that, compared to the non-immunosuppressed cohort, pulmonary infections in KTRs present a distinct pathogen spectrum dominated by viruses and opportunistic fungi, with a higher prevalence of co-infections. Furthermore, mNGS demonstrated significantly superior sensitivity over conventional methods in identifying these complex pathogens. Building on these results, our study further explored the clinical utility of non-invasive sputum specimens for diagnosing pulmonary infections in KTRs, providing new evidence to support the optimization of clinical diagnostic pathways.

First, our study confirms that the immunosuppressive state is a key determinant shaping the pathogen spectrum in KTRs with pulmonary infections. While the non-immunosuppressed control group exhibited a bacteria-dominant pathogen profile, the spectrum in KTRs was characterized by viruses (e.g., CMV, EBV) and opportunistic pathogens (e.g., *Pneumocystis jirovecii*). This finding is consistent with previous reports on infections in solid organ transplant recipients (Fishman, 2007; Pata et al., 2024; Ye et al., 2024). The underlying mechanism primarily involves long-term immunosuppressant use. Agents such as tacrolimus inhibit the calcineurin-NFAT signaling pathway, which in turn impairs T-cell function and cytokine production. This impairment restricts interleukin-2 (IL-2) release, directly weakening immune control over latent viruses (Torac et al., 2014). This immunological impairment explains our finding that members of the Herpesviridae family (CMV, EBV, HHV) collectively accounted for 25% of viral detections, a result that aligns with previous studies (Martín-Gandul et al., 2014; Kotton et al., 2025). Notably, the 6% detection rate of SARS-CoV-2 further corroborates the report by Saleem et al. (2022), who reported high susceptibility to SARS-CoV-2 among KTRs. This observation reflects that compromised T-cell immunity also impairs the host’s ability to control RNA viruses (Bensaid et al., 2023). Moreover, long-term immunosuppression not only weakens the host’s control over latent viruses but also creates a permissive environment for a wide range of opportunistic pathogens (Jiang et al., 2024; Li et al., 2025). The reactivation of many viruses exerts significant “indirect effects” by modulating the host immune response, thereby exacerbating the overall state of immunosuppression. The high detection rate of *Pneumocystis jirovecii* pneumonia (PJP), a representative opportunistic fungus, stems from this phenomenon (Marinaki et al., 2021). This is attributable to two key factors: first, KTRs exhibit reduced numbers and impaired function of CD4+ T cells, which directly compromises the activation of alveolar macrophages and their ability to clear *Pneumocystis jirovecii*; second, long-term oral corticosteroid use further suppresses local inflammatory responses, creating a microenvironment conducive to opportunistic fungal proliferation (Kovacs and Masur, 2000). The convergence of core immunosuppression, indirect effects of viral reactivation, and other clinical factors likely forms a “virus-opportunistic infection” vicious cycle (Fishman, 2007). This explains why the co-infection rate in KTRs in our study was as high as 67.57%, far exceeding that of the non-immunosuppressed group, which is consistent with prior research (Jiao et al., 2022). It is this complexity that often renders conventional diagnostic methods ineffective in identifying the causative pathogens in KTRs, frequently leading to empirical treatment failure (Li et al., 2022; Qin et al., 2022). In the present study, the detection rate of mycobacterial isolates was significantly higher in the non−immunocompromised group than in the KTR group (12.81% vs 2.00%). This discrepancy can be explained by two key factors: First, all kidney transplant recipients in our center underwent standardized pre-transplant latent tuberculosis infection screening and prophylaxis according to international and Chinese guidelines, which significantly reduces the risk of active tuberculosis. In contrast, non-immunosuppressed patients did not receive such routine screening or prophylaxis. Second, KTRs with mycobacterial infection often present with disseminated disease, which was excluded by our study design, further lowering the detection rate in the KTRs group. The striking difference in *Mycobacterium* detection rates further supports that standardized pre-transplant latent tuberculosis infection screening and prophylaxis effectively reduce the risk of active mycobacterial infection after kidney transplantation. Furthermore, we also analyzed and summarized the differences in pathogens detected by mNGS between BALF and sputum specimens in KTRs. This discrepancy may be mainly attributed to two factors. First, differences in the biological characteristics of the specimens themselves. BALF is directly collected from the alveolar spaces of the lower respiratory tract, which can directly reflect the pathogen composition in the infected lung parenchyma, thus conferring higher sensitivity for opportunistic pathogens mainly colonizing alveolar epithelial cells. Sputum specimens, however, are more susceptible to contamination by upper respiratory tract microbiota, resulting in a higher bacterial detection rate. Second, baseline differences existed between the two groups of patients. As shown in **Table 4**, the prevalence of comorbid heart disease was significantly higher in the sputum group, and most patients in this group were critically ill and unable to tolerate bronchoscopy. Differences in the course of infection and immunosuppressive status compared with the BALF group may also contribute to the variations in pathogen detection rates.

Second, this study demonstrates the superior diagnostic utility of mNGS for pulmonary infections in KTRs. Our data show extremely low concordance between mNGS and conventional methods (Kappa < 0.2), with mNGS successfully identifying pathogens in the vast majority of cases that tested negative by conventional tests. This finding underscores the inherent limitations of conventional tests—such as long turnaround times, low positivity rates, and particularly poor sensitivity for viruses and atypical pathogens—that render them ill-suited for diagnosing KTRs, who present with complex pathogen spectra (Zheng et al., 2021). As a culture-independent and unbiased detection technology, mNGS provides rapid and comprehensive etiological evidence. This evidence enables clinicians to shift from broad-spectrum empirical therapy to precise, targeted antimicrobial treatment, which holds immense value for improving patient outcomes and reducing unnecessary antibiotic use (Qi et al., 2023; Chen et al., 2025). However, a negative mNGS result does not exclude infection. Comprehensive evaluation and further evidence are still warranted, particularly in cases with high clinical suspicion of infection. In KTRs cohort, a total of 4 patients had negative mNGS results but were still clinically suspected of pulmonary infection (3 in the BALF subgroup and 1 in the sputum subgroup). For these patients, we adopted a comprehensive clinical evaluation strategy: treatment was guided by combining clinical symptoms, chest imaging findings, conventional etiological test results and multidisciplinary consultations (urology/transplantation, respiratory medicine, and infectious diseases), and empirical antimicrobial therapy was administered according to the latest clinical guidelines for pulmonary infection in kidney transplant recipients. The 1 patient in the sputum subgroup received maintenance of the original antimicrobial regimen; among the 3 patients in the BALF subgroup, 1 case received de-escalation therapy and 2 cases received maintenance of the original regimen, and all patients achieved favorable clinical improvement.

Finally, the most clinically significant finding of our study is the good diagnostic performance of sputum mNGS in KTRs. Our retrospective analysis revealed that the prevalence of comorbid heart disease in the sputum group (52.5%) was substantially higher than in the BALF group (2.7%). This observation may suggest that clinicians prudently select BALF, the “gold standard” for lower respiratory tract infections, for patients who are more stable and at lower risk, given the discomfort and cardiovascular stress associated with this invasive procedure (Shi et al., 2023). Consequently, patients with high-risk factors who cannot tolerate invasive procedures are more likely to undergo non-invasive sputum mNGS (Zhou et al., 2023). Of course, some patients may be unable to produce a sputum sample, necessitating the use of other appropriate specimens. Therefore, the true value of our study lies in revealing the real-world clinical utility of these two sampling strategies. Our study found that sputum mNGS exhibited excellent diagnostic efficacy, a result that challenges the conventional wisdom that invasive sampling is obligatory. Several factors may underlie this observation. First, in clinical practice, patients with productive cough are more likely to have sputum collected for mNGS. The higher prevalence of productive cough in our sputum group may have yielded higher-quality specimens that were more representative of lower respiratory tract pathogens. Second, the reduced time interval between admission and sputum collection (median 1 vs. 2 days) may have permitted sampling during the early stage of infection, when pathogen nucleic acid loads are higher. In contrast, BALF collection may be delayed due to patient intolerance, resulting in sampling in the mid-to-late stages of infection. Additionally, if the lavage procedure fails to cover all infected sites, its diagnostic efficacy may also be reduced (He et al., 2020; Yin et al., 2022). Although a major concern with sputum mNGS is interference from oral commensals, our finding that pulmonary infections in KTRs are dominated by a unique spectrum of viruses and opportunistic fungi allows clinicians to more effectively discern true pathogens when interpreting results in the context of clinical and radiological findings. Furthermore, the high sensitivity of mNGS may compensate for potentially lower pathogen concentrations in sputum compared to BALF (Jin et al., 2025). Notably, The baseline data of this study showed no significant differences in immunosuppressive status and transplant renal function between the sputum group and the BALF group (all P>0.05), effectively eliminating the confounding effects of these two key clinical factors on the comparison of mNGS detection performance. Meanwhile, serum creatinine levels remained stable in kidney transplant recipients before and after antimicrobial therapy, and tacrolimus trough concentrations were appropriately adjusted during infection, which is consistent with the standardized clinical management of kidney transplant recipients with pulmonary infection. Crucially, our data show no significant differences between the two specimen types in guiding therapy adjustments or in final patient outcomes, while sputum mNGS demonstrated high positive detection rates and concordance with clinical diagnosis. This result provides further evidence that for critically ill patients who cannot tolerate BALF, sputum mNGS represents an accessible and effective diagnostic strategy. The practical challenge of patients being unable to produce sputum can be addressed via techniques such as hypertonic saline induction.

This study has several limitations. First, its single-center, retrospective design is susceptible to selection bias. Although we attempted to mitigate this through statistical adjustments, a multivariable analysis to fully control for confounding factors was not performed. Second, due to cost and ethical considerations, we did not perform paired BALF and sputum mNGS on the same patients, nor did we assess the cost-effectiveness of sputum mNGS from a health economics perspective. In addition, restricted by real-world clinical diagnostic routines, we were unable to enroll a valid non-immunosuppressed control cohort with sputum mNGS testing. And, detailed data regarding previous pulmonary infections in the non-immunosuppressed group were not collected in this study, as these patients lack long-term regular follow-up and systematic medical records, making reliable retrospective collection unfeasible. Furthermore, this was an exploratory *post-hoc* analysis without a pre-calculated sample size; a multicenter, paired-sample cohort study is warranted. We also did not perform subgroup analyses to explore the impact of factors such as immunosuppressive regimens or post-transplant duration on the pathogen spectrum. Finally, mNGS itself cannot distinguish between colonization and infection, requiring further validation with clinical parameters.

In conclusion, the distinct pathogen spectrum and high rate of co-infection in KTRs suggest that initial empirical antimicrobial therapy should provide broad-spectrum coverage. On this basis, potential pathogenic pathogens should be identified as early as possible, and a comprehensive clinical evaluation should be rigorously integrated to differentiate colonization from true infection. As a non-invasive and easily accessible specimen, sputum exhibited good diagnostic performance, offering a new clinical option, especially for unstable patients unable to undergo immediate bronchoscopy or in resource-limited settings. From a health economics perspective, the non-invasive nature, repeatability, and shorter turnaround time of sputum testing suggest it may have a favorable cost-effectiveness ratio. The good diagnostic performance of sputum mNGS in this study raises a critical question for future clinical practice: what role can it play? Our findings suggest that for this special population of KTRs, sputum testing may be more than a last resort for critically ill patients. It has the potential to serve as an important ancillary or even alternative, complementing or partially replacing invasive BALF sampling. Future research should prioritize prospective, paired-sample studies to formally evaluate the diagnostic concordance between sputum and BALF mNGS. If our preliminary findings are confirmed, it could optimize current diagnostic pathways, establishing a less invasive and more efficient strategy that would significantly benefit patients, clinicians, and the healthcare system.

## 5 Conclusion

In summary, pulmonary infections in kidney transplant recipients present a distinct pathogen spectrum dominated by viruses and opportunistic fungi. Our study not only confirms that non-invasive sputum mNGS is an effective diagnostic tool for high-risk patients who cannot tolerate invasive procedures but also suggests its potential as a reliable alternative to BALF in the broader KTRs population. Future large-scale, paired-sample prospective studies are warranted to validate these findings and promote the standardized use of sputum mNGS in clinical practice.

## Data Availability

The original contributions presented in the study are included in the article/supplementary material. Further inquiries can be directed to the corresponding author.
